# Association of gallstone and polymorphisms of UGT1A1*27 and UGT1A1*28 in patients with hepatitis B virus-related liver failure

**DOI:** 10.1515/med-2022-0549

**Published:** 2022-09-06

**Authors:** Haiyan Zhuo, Jinhai Fan, Bifeng Zhang, Yixian Shi, Liqing Zheng, Yihong Chai, Lvfeng Yao

**Affiliations:** Department of Hepatology, Mengchao Hepatobiliary Hospital of Fujian Medical University, No. 312 Xihong Road, Fuzhou, Fujian, 350025, P. R. China; Department of Hepatology, Mengchao Hepatobiliary Hospital of Fujian Medical University, Fuzhou, Fujian, 350025, P. R. China; Department of Gastroenterology, Quanzhou First Hospital, Quanzhou, Fujian, 362000, P. R. China

**Keywords:** *UGT1A1*, gallstone, liver failure, hepatitis B virus, single-nucleotide polymorphism

## Abstract

Genetic variation in UDP-glucuronosyltransferase 1A1 gene (UGT1A1) is a lithogenic risk factor for gallstone formation. This study aimed to assess genotype and allele frequencies of common UGT1A1 variants in patients with gallstone and hepatitis B virus (HBV)-related hepatic failure. This study enrolled 113 healthy individuals (CTRL), 54 patients with HBV infection (HBV), 134 patients with gallstone-free hepatic failure and HBV infection, and 34 patients with gallstone-related hepatic failure and HBV infection (GRHF). Peripheral venous blood samples were collected for genomic DNA isolation. Polymerase chain reaction amplification was carried out for UGT1A1, followed by direct sequencing. Analysis for genotype and allele frequencies of UGT1A1 variants (UGT1A1*6, UGT1A1*27, UGT1A1*28, and UGT1A1*60) was performed. The allele distributions of the four groups did not deviate from Hardy–Weinberg equilibrium. Allele (A) and genotype (CA) frequency distributions of UGT1A1*27 were significantly different between GRHF and CTRL, or between GRHF and HBV. GRHF and CTRL exhibited significant differences in allele (A) and genotype (CA) frequency distributions of UGT1A1*28. Linkage disequilibrium analysis suggested that haplotype G-G-[TA]7-T may be associated with gallstone in HBV-related hepatic failure. Our data reveal that UGT1A1*27 and UGT1A1*28 variants are significantly observed in patients with GRHF compared to healthy individuals.

## 1 Introduction

Chronic hepatitis B virus (HBV) infection frequently causes severely progressive hepatic diseases, including fibrosis, cirrhosis, hepatocellular carcinoma, and hepatic failure [1,2,3]. There are at least 292 million chronical carriers of HBV accounting for 3.9% of the world’s population, leading to 880,000 deaths from liver failure due to cirrhosis annually [4,5]. Available evidence shows that chronic infection with HBV is the most common causative factor of liver failure in China [6,7,8]. Recent studies have provided evidence on the association between the risk of gallstones and HBV infection [9,10]. Most notably, patients with cirrhosis and gallstone were detected with high levels of total bilirubin, direct bilirubin and indirect bilirubin compared with cirrhosis patients without gallstone [11]. The elevated bilirubin exacerbates liver failure for patients with HBV infection and is a valuable marker for predicting prognosis for patients with cirrhosis and liver failure [12,13].

UDP-glucuronosyltransferase (UGT) comprises a superfamily of enzymes that catalyze the glucuronidation reaction [14,15,16]. UDP-glucuronosyltransferase 1A1 gene (*UGT1A1*) is determined as the only related enzyme implicated in the glucuronidation of bilirubin that is a degradation product under a normal catabolic condition [17,18,19]. The deficiency of UGT1A1 enzyme results in serve unconjugated hyperbilirubinemia, appearing to be a risk factor for gallstone formation in Jamaican patients with sickle cell disease [20]. More importantly, UGT1A1 mutation is a pathogenic risk factor for cholelithiasis, given that UGT1A1 defects lead to bile acid malabsorption accompanied by enhanced bilirubin uptake and the development of hyperbilirubinemia [21,22,23]. Recently, allelic variants of *UGT1A1* gene have gained major attentions since its polymorphism is associated with bilirubin levels and liver function in HBV-positive or HCV-positive carriers [24,25].

Historical research uncovers polymorphisms of *UGT1A1* in the promoter or exon 1 regions contribute to unconjugated hyperbilirubinemia, which may be the source of gallstone formation, with UGT1A1*6 (c.211G>A, rs4148323, p.Gly71Arg), UGT1A1*27 (c.686C>A, rs35350960, p.Pro229Glu), UGT1A1*28 ([TA]6>[TA]7, rs3064744, rs4124874), and UGT1A1*60 (c.-3279T>G) mostly reported [26,27,28,29]. It was identified HBV-positive carriers of heterozygosis or homozygosis for UGT1A1*60; polymorphism analysis suggested that these individuals are more susceptible to cancer [30]. The most common polymorphism of *UGT1A1* is an additional TA repeat in the TATA box region of the promoter, while the predominant variation in Asians is a missense mutation, c211G>A (p.G71R) [31,32]. The genetic mutation UGT1A1*27 (c.686C>A) contributes to severe neonatal hyperbilirubinemia in Jaundiced neonates [33]. However, the information is lacking about the important *UGT1A1* gene variations in gallstone-related liver failure caused by HBV infection. The aim of this study was to analyze the association between *UGT1A1* polymorphisms (UGT1A1*6, UGT1A1*27, UGT1A1*28, and UGT1A1*6) and gallstone in patients with hepatitis B-related liver failure.

## 2 Materials and methods

### 2.1 Subjects

This study comprised a hospital-based study of 335 subjects, including 113 healthy individuals (CTRL), 54 patients with HBV infection and without gallstone and hepatic failure (HBV), 134 patients with gallstone-free hepatic failure and HBV infection (GFHF), and 34 patients with gallstone-related hepatic failure and HBV infection (GRHF). Adult patients with hepatic failure received general internal medicine treatment and were treated with plasma exchange-centered artificial liver support system at our hospital from June 2015 to September 2017. Clinical information including basic characteristics (gender, age, and complications) and biochemical examinations was recorded. The research related to human use has been complied with all the relevant national regulations and institutional policies and in accordance with the tenets of the Helsinki Declaration and has been approved by the Ethics Committee of our Hospital (No. 2021-019-01). Informed consent has been obtained from all individuals included in this study.

### 2.2 DNA isolation and data analysis

**Figure 1:**
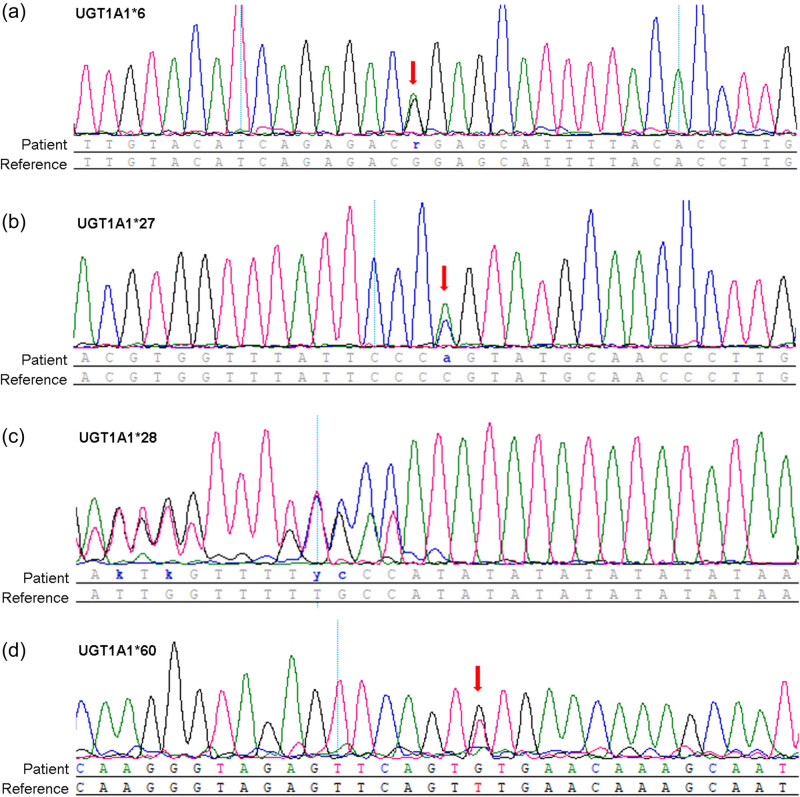
Whole-exome seuqencing variants from *UGT1A1*. Electropherograms exhibit the variant positions, (a) UGT1A1*6 (rs4124874, c.-3279T>G, p.Gly71Arg), (b) UGT1A1*27 (c.686C>A, rs35350960, p.Pro229Glu), (c) UGT1A1*28 (rs3064744, [TA]6>[TA]7), and (d) UGT1A1*60 (rs4124874, c.-3279T>G) marked by red arrows.

Peripheral venous blood samples were taken in 10–12 h fasting status in the morning and stored at −20°C in ethylene diamine tetra-acetic acid-containing vacutainer. Genomic DNA was extracted using the Genomic DNA Isolation Kit (Tiangen). *UGT1A1* was amplified by PCR with 30 ng genomic DNA, 0.5 μL specific primers (10 pmol) (Table 1), 2 μL dNTP (2.5 mmol), and 1 μL rTaq. PCR amplification was carried out on an ABI 9700 PCR thermal cycler (ABI). The reaction procedure was initial denaturation at 95°C for 5 min, followed by 35 cycles of denaturation at 95°C for 30 s, primer annealing at 60°C for 30 s, primer extension at 72°C for 1 min, and final extension at 72°C for 5 min. The amplified product was separated using 1.5% agarose gel electrophoresis. The collected PCR products were purified with the MagNA Pure LightCycler 32 instrument (Roche Applied Science, Indianapolis, IN, USA). Genotyping of four SNPs in the promoter and exon regions of *UGT1A1*, namely UGT1A1*6 (rs4148323), UGT1A1*27 (rs35350960), UGT1A1*28 (rs3064744), and UGT1A1*60 (rs4124874) was determined by the sequencing of PCR products. The purified PCR amplicons were sequenced by BioSune Tech Co., Ltd. (Shanghai, China). After removal of the primer regions, all sequences were aligned with DNAstar’s SeqMan software (DNAStar, Madison, WI, USA). Representative electropherograms are shown in Figure 1.

**Table 1:** Sequence of primers for targeting genomic amplicon sequencing for *UGT1A1*

Sites	ID	Primer sequences (5′−3′)	
		Forward	Reverse
Enhancer	UGT1A10628-PBERM	AGGTGTAATGAGGATGTGTT	CTCTTACCCTCTAGCCATTC
TATA box	UGT1A10628-TATABOX	CCAGTTCAACTGTTGTTGCC	TCCTGCCAGAGGTTCGCCCT
Exon1a	70316-UGT1A1-1a	TGAACTCCCTGCTACCTTTGT	CAGTGGGCAGAGACAGGTAC
Exon1b	70316-UGT1A1-1b	TCTGCTATGCTTTTGTCTGGC	TGCCAAAGACAGACTCAAACC
Exon2	70316-UGT1A1-2	CAAACACGCATGCCTTTAATCA	GGATTAATAGTTGGGAAGTGGCA
Exon3	70316-UGT1A1-3	CCAGTCCTCAGAAGCCTTCA	GCAATGTAGGATATGTTGGCCA
Exon4	70316-UGT1A1-4	TGGCCAACATATCCTACATTGC	AACAACGCTATTAAATGCTACGT
Exon5	70316-UGT1A1-5	ACAGGGCAAGACTCTGTATCT	CCTGATCAAAGACACCAGAGG

### 2.3 Statistical analysis

Serum levels of total bilirubin, direct bilirubin, and indirect bilirubin were presented as the median and interquartile range (25th percentile to 75th percentile), and multiple comparisons were carried out using one-way ANOVA corrected by Tukey test. Hardy−Weinberg equilibrium, genotype distribution frequency, allele frequency, linkage disequilibrium (LD), and haplotype distribution were analyzed according to Shi’s method [34]. The normalizing coefficient LD (|*D*′|) and the square of correlation coefficient between pairs of loci (*r* ^2^) were calculated for all pairs of alleles (c.-3279T>G, [TA]6>[TA]7, c.211G>A, and c.686C>A). |*D*′| and *r* ^2^ of 1 correspond to complete LD, while 0 presents no LD. |*D*′| > 0.8 and *r* ^2^ > 0.8 indicate a high extent of LD. Differences in the proportion of four SNPs between HBV and CTRL, GRHF and CTRL, GFHF and HBV, GRHF and HBV, or GRHF and GFHF were analyzed using Pearson’s chi-square test with Yates’ continuity correction or Fisher’s exact test. Fisher’s exact test was used for the calculation of 95% confidence interval of the difference between proportions with less than five subjects.

## 3 Results

### 3.1 Bilirubin levels were different among the CTRL, HBV, GFHF and GRHF patients

**Figure 2:**
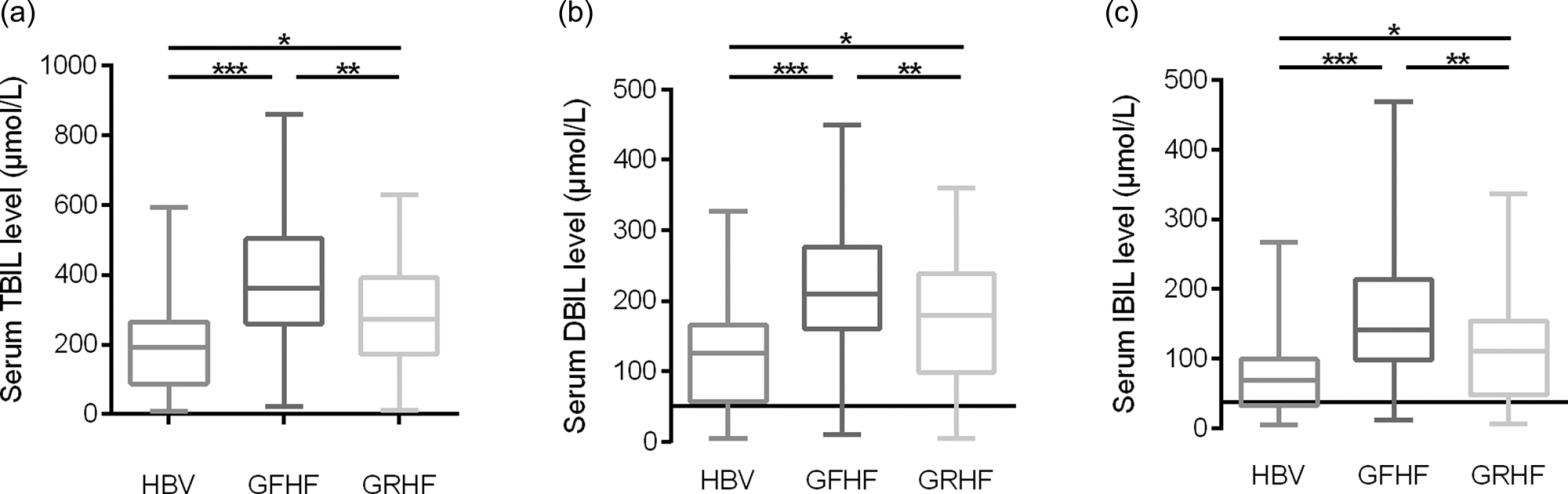
Serum levels of (a) TBIL, (b) DBIL, and (c) IBIL increased in patients with HBV infection, GFHF and GRHF. Boxplots represted the median, 25th percentile, 75th percentile, and error bars. TBIL, total bilirubin; DBIL, direct bilirubin; IBIL, indirect bilirubin. HBV, hepatitis B virus. CTRL group, healthy individuals without HBV infection, gallstone, or hepatic failure; HBV group, patients with HBV infection; GFHF, patients with gallstone-free hepatic failure and HBV infection; GRHF, patients with gallstone-related hepatic failure and HBV infection. CTRL (*n* = 113), HBV (*n* = 54), GFHF (*n* = 134), and GRHF (*n* = 34). *p < 0.05, **p < 0.01, and ***p < 0.001 by one-way ANOVA corrected by Tukey test.

Serum total bilirubin levels were significantly higher in patients with HBV infection (median 192.0 μmol/L; IQR 86.75–265.4) as defined by a serum total bilirubin ranging 0–26 μmol/L compared with healthy individuals (Figure 2). Direct bilirubin (median 125.0 μmol/L; IQR 56.50–166.6; *p* < 0.0001) and indirect bilirubin (median 68.40 μmol/L; IQR 32.75–99.55) were higher in HBV patients compared with healthy participants (direct bilirubin ranging 0–8 μmol/L; indirect bilirubin ranging 0.5–20.0 μmol/L). GFHF patients possessed higher levels of TBIL (median 362.4 μmol/L, IQR 259.9–503.0), DBIL (median 209.3 μmol/L, IQR 160.8–276.5), and IBIL (median 141.6 μmol/L, IQR 97.78–214.5) than HBV patients (TBIL: median 192.0 μmol/L, IQR 86.75–265.4, *p* < 0.0001; DBIL: median 125.0 μmol/L, IQR 56.50–166.6, *p* < 0.0001; IBIL: median 68.40 μmol/L, IQR 32.75–99.55, *p* < 0.0001). Consistently, patients with GRHF had higher levels of TBIL (median 362.4 μmol/L; IQR 259.9–503.0), DBIL (median 209.3 μmol/L; IQR 160.8–276.5), and IBIL (median 141.6 μmol/L; IQR 97.78–214.5) than HBV patients (TBIL, *p* = 0.0266; DBIL, *p* = 0.0326; IBIL, *p* = 0.0391). Of note, TBIL, DBIL, and IBIL levels differed significantly between GFHF patients and GRHF patients (TBIL, *p* = 0.0019; DBIL, *p* = 0.0026; IBIL, *p* = 0.0032).

### 3.2 UGT1A1 variants and Hardy–Weinberg equilibrium analysis

In the present study, the subjects in the CTRL group comprised 26.5% UGT1A1*6 (rs4124874, c.-3279T>G, p.Gly71Arg), 15.9% UGT1A1*28 (rs3064744, [TA]6>[TA]7), and 59.3% UGT1A1*60 (rs4124874, c.-3279T>G) (Table 2). HBV-infected individuals were detected with 29.6% UGT1A1*6, 24.1% UGT1A1*28, and 53.7% UGT1A1*60. UGT1A1*6, UGT1A1*27, UGT1A1*28, and UGT1A1*60 were noted in 34.3, 4.5, 26.1, and 56.0% GFHF patients, respectively. GRHF group consisted of 32.4% UGT1A1*6, 8.8% UGT1A1*27, 35.3% UGT1A1*28, and 52.9% UGT1A1*60. The frequency of UGT1A1*27 and UGT1A1*28 in GRHF patients is significantly higher than that of healthy participants (*p* < 0.05). The odd ratios of UGT1A1*27 and UGT1A1*28 were 25.22 [1.27, 502] and 2.88 [1.21, 6.84] when the CTRL group was compared the GRHF group (Table S1). These results indicated that UGT1A1*27 and UGT1A1*28 showed a strong association with GRHF. The observed genotype distributions of the CTRL, HBV, GFHF, and GRHF subjects were not obviously different from the values computed by Fisher’s exact test (*p* > 0.05), suggesting that the allele distributions of the CTRL, HBV, GFHF, and GRHF groups did not deviate from Hardy–Weinberg equilibrium (Table 3).

**Table 2:** Odds ratio and 95% CI for gallbladder stone-related hepatic failure associated with *UGT1A1* (NM_000463) variants

	Polymorphisms			
	UGT1A1*6	UGT1A1*27	UGT1A1*28	UGT1A1*60
Variant	c.211G>A	c.686C>A	[TA]6>[TA]7	c.-3279T>G
Amino acid change	p.Gly71Arg	p.Pro229Glu	—	—
SNP ID	rs4148323	rs35350960	rs3064744	rs4124874
Location	Exon 1	Exon 1	Promoter	Promoter
Position	GRCh38.p13	GRCh38.p13	GRCh38.p13	GRCh38.p13
Type of variant	Missense	Missense	Upstream transcript	Upstream transcript
CTRL (*n*, freq)	30, 26.5%	0, 0.0%	18, 15.9%	67, 59.3%
HBV (*n*, freq)	16, 29.6%	0, 0.0%	13, 24.1%	29, 53.7%
GFHF (*n*, freq)	46, 34.3%	6, 4.5%	35, 26.1%	75, 56.0%
GRHF (*n*, freq)	11, 32.4%	3, 8.8%^a^	12, 35.3%^a^	18, 52.9%

UGT1A1, UDP-glucuronosyltransferase 1A1; SNP, single-nucleotide polymorphism; HBV, hepatitis B virus. *p*-values were calculated using Fisher’s exact test or Pearson’s chi-square test (^a^ *p* < 0.05 compared to CTRL group). CTRL group, healthy individuals without HBV infection, gallstone, or hepatic failure; HBV group, patients with HBV infection; GFHF, patients with gallstone-free hepatic failure and HBV infection; GRHF, patients with gallstone-related hepatic failure and HBV infection. CTRL (*n* = 113), HBV (*n* = 54), GFHF (*n* = 134), and GRHF (*n* = 34).

**Table 3:** Hardy–Weinberg equilibrium analysis

Polymorphisms	Fisher’s *p*-value			
	CTRL	HBV	GFHF	GRHF
UGT1A1*6	0.6559	0.7288	0.9341	0.4010
UGT1A1*27	1.0000	1.0000	0.7909	0.7878
UGT1A1*28	0.3577	0.9111	0.0821	0.7878
UGT1A1*60	0.8330	0.8382	0.4033	0.7296

The *p*-values of Fisher’s exact test or or Pearson’s chi-square test more than 0.05 meet the Hardy–Weinberg equilibrium. UGT1A1, UDP-glucuronosyltransferase 1A1; HBV, hepatitis B virus. CTRL group, healthy individuals without HBV infection, gallstone, or hepatic failure; HBV group, patients with HBV infection; GFHF, patients with gallstone-free hepatic failure and HBV infection; GRHF, patients with gallstone-related hepatic failure and HBV infection. CTRL (*n* = 113), HBV (*n* = 54), GFHF (*n* = 134), and GRHF (*n* = 34).

### 3.3 UGT1A1 polymorphism was associated with the development of GRHF

The differences in allele frequencies and genotype distribution of *UGT1A1* SNPs were analyzed between GRHF patients and GFHF patients. It was suggested that GRHF patients and CTRL patients exhibited significant differences in allele frequencies distributions of UGT1A1*27 (*p* = 0.0120) and UGT1A1*28 (*p* = 0.0129) (Table 4 and Table S2). There was significantly different in the distribution of genotype CA of UGT1A1*27 between the CTRL and GRHF group (*p* = 0.0115) or HBV and GRHF group (*p* = 0.0264). The distribution of genotype [TA]6[TA]7 of UGT1A1*28 was obviously different between the GRHF and CTRL group (*p* = 0.0448). In contrast, no significant difference in genotype distribution and allele frequencies of UGT1A1*6 and UGT1A1*60 was observed between the GRHF and CTRL group or the GRHF and HBV group (*p* > 0.05).

**Table 4:** Genotype distribution and allele frequencies of four SNPs in *UGT1A1* gene with the development of hepatic failure associated with gallbladder stone

Polymorphisms	Genotype (*n*, frequency)			Allele (*n*, frequency)	
UGT1A1*6	GG	GA	AA	G	A
CTRL (*n*, freq)	83, 73.5%	27, 23.9%	3, 2.7%	193, 85.4%	33, 14.6%
HBV (*n*, freq)	38, 70.4%	15, 27.8%	1, 1.9%	91, 84.3%	17, 15.7%
GFHF (*n*, freq)	88, 65.7%	41, 30.6%	5, 3.7%	217, 81.0%	51, 19.0%
GRHF (*n*, freq)	23, 67.6%	9, 26.5%	2, 5.9%	55, 80.9%	13, 19.1%

UGT1A1*27	CC	CA	AA	C	A
CTRL (*n*, freq)	113, 100%	0, 0.0%	0, 0.0%	226, 100.0%	0, 0.0%
HBV (*n*, freq)	54, 100%	0, 0.0%	0, 0.0%	108, 100.0%	0, 0.0%
GFHF (*n*, freq)	128, 95.5%	6, 4.5%	0, 0.0%	262, 97.8%	6, 2.2%
GRHF (*n*, freq)	31, 91.2%	3, 8.8%ab	0, 0.0%	65, 95.6%	3, 4.4%ab

UGT1A1*28	[TA]6[TA]6	[TA]6[TA]7	[TA]7[TA]7	[TA]6	[TA]7
CTRL (*n*, freq)	95, 84.1%	18, 15.9%	0, 0.0%	208, 92.0%	18, 8.0%
HBV (*n*, freq)	41, 75.9%	12, 22.2%	1, 1.9%	94, 87.0%	14, 13.0%
GFHF (*n*, freq)	99, 73.9%	35, 26.1%	0, 0.0%	233, 86.9%	35, 13.1%
GRHF (*n*, freq)	22, 64.7%	11, 32.4%a	1, 2.9%	55, 80.9%	13, 19.1%a

UGT1A1*60	TT	TG	GG	T	G
CTRL (*n*, freq)	46, 40.7%	53, 46.9%	14, 12.4%	145, 64.2%	81, 35.8%
HBV (*n*, freq)	25, 46.3%	23, 42.6%	6, 11.1%	73, 67.6%	35, 32.4%
GFHF (*n*, freq)	59, 44.0%	63, 47.0%	12, 9.0%	181, 67.5%	87, 32.5%
GRHF (*n*, freq)	16, 47.1%	14, 41.2%	4, 11.8%	46, 67.6%	22, 32.4%

*p*-values were calculated using Fisher’s exact test or or Pearson’s chi-square test (lowercase character “a” indicates *p* < 0.05 compared to the CTRL group, “b” indicating *p* < 0.05 compared to the HBV group). UGT1A1, UDP-glucuronosyltransferase 1A1; SNP, single-nucleotide polymorphism; HBV, hepatitis B virus. CTRL group, healthy individuals without HBV infection, gallstone, or hepatic failure; HBV group, patients with HBV infection; GFHF, patients with gallstone-free hepatic failure and HBV infection; GRHF, patients with gallstone-related hepatic failure and HBV infection. CTRL (*n* = 113), HBV (*n* = 54), GFHF (*n* = 134), and GRHF (*n* = 34).

### 3.4 Association between UGT1A1 haplotypes and development of GRHF

The LD pattern across the multiple SNPs of *UGT1A1* is shown in Table 5. When CRHF was compared with CTRL, UGT1A1*60 and UGT1A1*6 (|*D*′| > 0.8, *r* ^2^ = 0.100) or stie 4 and UGT1A1*28 (|*D*′| = 0.782, *r* ^2^ = 0.134) showed moderate pairwise LD. Moderate pairwise LD was observed between UGT1A1*60 and UGT1A1*6 (|*D*′| > 0.8, *r* ^2^ = 0.094, HBV vs CTRL; |*D*′| > 0.8, *r* ^2^ = 0.098, GRHF vs HBV; |*D*′| > 0.8, *r* ^2^ = 0.113, GRHF vs GFHF), UGT1A1*60 and UGT1A1*28 (|*D*′| > 0.8, *r* ^2^ = 0.147, HBV vs CTRL; |*D*′| = 0.790, *r* ^2^ = 0.236, GRHF vs HBV; |*D*′| > 0.8, *r* ^2^ = 0.271, GRHF vs GFHF). In comparison between the HBV and CTRL group, UGT1A1*28 and UGT1A1*27 (|*D*′| = 0.8, *r* ^2^ = 0.108), UGT1A1*60 and UGT1A1*6 (|*D*′| > 0.8, *r* ^2^ = 0.106), or UGT1A1*60 and UGT1A1*28 (|*D*′| > 0.8, *r* ^2^ = 0.266) showed moderate pairwise LD. Low pairwise LD was found within UGT1A1*6, UGT1A1*27, UGT1A1*28, and UGT1A1*60 (|*D*′| < 0.8 or *r* ^2^ ranging 0.000–0.082). As a result, LD analysis showed the four SNPs were not significantly associated with each other. There was no evidence of apparent LD. Thus, it was likely that the four SNPs independently contribute to the association.

**Table 5:** Linkage disequilibrium between different SNPs of *UGT1A1* gene

	GRHF vs CTRL			HBV vs CTRL			GFHF vs HBV			GRHF vs HBV			GRHF vs GFHF		
|*D*′|	Site 2	Site 3	Site 4	Site 2	Site 3	Site 4	Site 2	Site 3	Site 4	Site 2	Site 3	Site 4	Site 2	Site 3	Site 4
Site 1	0.024	0.998	0.999	0.000	1.000	0.999	0.183	0.998	1.000	0.036	0.997	0.999	0.325	0.997	1.000
Site 2	—	0.255	0.487	—	0.000	0.000	—	1.000	0.998	—	0.213	0.507	—	0.704	0.790
Site 3	—	—	0.782	—	—	0.859	—	—	0.923	—	—	0.790	—	—	0.883

*r* ^2^	Site 2	Site 3	Site 4	Site 2	Site 3	Site 4	Site 2	Site 3	Site 4	Site 2	Site 3	Site 4	Site 2	Site 3	Site 4
Site 1	0.000	0.022	0.100	0.000	0.019	0.094	0.000	0.033	0.106	0.000	0.037	0.098	0.001	0.039	0.113
Site 2	—	0.006	0.005	—	0.000	0.000	—	0.108	0.034	—	0.004	0.009	—	0.082	0.036
Site 3	—	—	0.134	—	—	0.147	—	—	0.266	—	—	0.236	—	—	0.271

SNP, single-nucleotide polymorphism; UGT1A1, UDP-glucuronosyltransferase 1A1; HBV, hepatitis B virus. |*D*′|, the normalizing coefficient linkage disequilibrium; *r* ^2^, the square of correlation coefficient between pairs of loci; |*D*′| and *r* ^2^ of 1 correspond to complete LD, while 0 presents no LD. CTRL group, healthy individuals without HBV infection, gallstone, or hepatic failure; HBV group, patients with HBV infection; GFHF, patients with gallstone-free hepatic failure and HBV infection; GRHF, patients with gallstone-related hepatic failure and HBV infection. CTRL (*n* = 113), HBV (*n* = 54), GFHF (*n* = 134), and GRHF (*n* = 34).

Because all the four SNPs were within the *UGT1A1* region, we focused on a haplotype analysis. The distribution of haplotypes of *UGT1A1* polymorphisms (UGT1A1*6, UGT1A1*27, UGT1A1*28, and UGT1A1*60) in the disease and control groups is presented in Table 6. The results suggested that the haplotype G-C-[TA]6-G was significantly associated with healthy people (*p* < 0.05) rather than patients with GRHF, and odds ratio was 0.472. The haplotype G-C-[TA]7-T was distinctly related with GRHF patients compared with GFHF patients (*p* < 0.05), showing an increased risk of GRHF (OR = 7.616; 95% CI 0.887–65.411).

**Table 6:** Haplotype distribution of *UGT1A1* polymorphisms (UGT1A1*6, UGT1A1*27, UGT1A1*28, and UGT1A1*60) among different groups

Haplotype	GRHF (*n*, freq)	CTRL (*n*, freq)	*p*-value	OR [95% CI]
G C [TA]6G	10.39, 15.3%	64.93, 28.7%	0.0388	0.472 [0.229, 0.973]
G C [TA]7G	9.60, 14.1%	16.07, 7.1%	0.0551	2.264 [0.965, 5.310]
A C [TA]6T	11.99, 17.6%	33.00, 14.6%	0.4498	1.323 [0.639, 2.738]
G C [TA]6T	30.6, 45.0%	110.07, 48.7%	0.8215	0.938 [0.540, 1.631]
G C [TA]7T	2.39, 3.5%	1.93, 0.9%	0.0972	4.434 [0.652, 30.177]

Haplotype	HBV (*n*, freq)	CTRL (*n*, freq)	*p*-value	OR [95% CI]
A C [TA]6T	16.99, 15.7%	33.00, 14.6%	0.7740	1.098 [0.581, 2.075]
G C [TA]6G	22.38, 20.7%	64.93, 28.7%	0.1238	0.651 [0.376, 1.127]
G C [TA]6T	54.62, 50.6%	110.07, 48.7%	0.7192	1.088 [0.686, 1.726]
G C [TA]7G	12.61, 11.7%	16.07, 7.1%	0.1596	1.737 [0.799, 3.775]

Haplotype	GFHF (*n*, freq)	HBV (*n*, freq)	*p*-value	OR [95% CI]
A C [TA]6T	50.94, 19.0%	16.99, 15.7%	0.4196	1.281 [0.701, 2.339]
G C [TA]6G	53.31, 19.9%	22.38, 20.7%	0.9068	0.967 [0.555, 1.685]
G C [TA]6T	128.75, 48.0%	54.62, 50.6%	0.7475	0.929 [0.592, 1.457]
G C [TA]7G	27.69, 10.3%	12.61, 11.7%	0.7368	0.886 [0.437, 1.797]

Haplotype	GRHF (*n*, freq)	HBV (*n*, freq)	*p*-value	OR [95% CI]
A C [TA]6T	11.99, 17.6%	16.99, 15.7%	0.6433	1.212 [0.537, 2.732]
G C [TA]6G	10.39, 15.3%	22.38, 20.7%	0.4414	0.728 [0.324, 1.637]
G C [TA]6T	30.62, 45.0%	54.62, 50.6%	0.6591	0.871 [0.470, 1.612]
G C [TA]7G	9.60, 14.1%	12.61, 11.7%	0.5560	1.311 [0.532, 3.232]
G C [TA]7T	2.39, 3.5%	1.39, 1.3%	0.2974	2.931 [0.354, 24.290]

Haplotype	GRHF (*n*, freq)	GFHF (*n*, freq)	*p*-value	OR [95% CI]
A C [TA]6T	11.99, 17.6%	50.94, 19.0%	0.8554	0.937 [0.467, 1.883]
G C [TA]6G	10.39, 15.3%	53.31, 19.9%	0.4271	0.745 [0.360, 1.543]
G C [TA]6T	30.62, 45.0%	128.75, 48.0%	0.7701	0.922 [0.535, 1.589]
G C [TA]7G	9.60, 14.1%	27.69, 10.3%	0.3403	1.466 [0.665, 3.232]
G C [TA]7T	2.39, 3.5%	1.31, 0.5%	0.0301	7.616 [0.887, 65.411]

All those frequency <0.03 was ignored in analysis; *P*-values were calculated using Fisher’s exact test or Pearson’s chi-square test. UGT1A1, UDP-glucuronosyltransferase 1A1; OR, odds ratio; CI, confidence interval; SNP, single-nucleotide polymorphism; UGT1A1, UDP-glucuronosyltransferase 1A1; HBV, hepatitis B virus. CTRL group, healthy individuals without HBV infection, gallstone, or hepatic failure; HBV group, patients with HBV infection; GFHF, patients with gallstone-free hepatic failure and HBV infection; GRHF, patients with gallstone-related hepatic failure and HBV infection. CTRL (*n* = 113), HBV (*n* = 54), GFHF (*n* = 134), and GRHF (*n* = 34).

## 4 Discussion

Genetic variation in *UGT1A1* underlies the development of unconjugated hyperbilirubinemia that is a lithogenic risk factor for gallstone formation in multiple diseases, such as sickle cell disease [20], cystic fibrosis [21], and pigmentous gallstones [22]. Here, we revealed the association between *UGT1A1* polymorphisms (UGT1A1*6, UGT1A1*27, UGT1A1*28, and UGT1A1*60) and GRHF. Patients in HBV, GFHF, and GRHF groups showed higher levels of total bilirubin, direct bilirubin, and indirect bilirubin. UGT1A1*27 and UGT1A1*28 showed a strong connection with GRHF. The haplotype G-C-[TA]7-T was distinctly related with GRHF patients compared with GFHF patients.

UGT1A1*6 polymorphism is frequent in neonates with severe hyperbilirubinemia in the Chaozhou region of southern China [26]. The compound heterozygous UGT1A1*6 and UGT1A1*28 are major genotypes associated with the high risks of hyperbilirubinemia in Chinese Han people [35]. UGT1A1*6 variants contribute to disordered bilirubin that results the clinical phenotype of neonatal hyperbilirubinemia [36]. However, the effect of UGT1A1*6 gene polymorphisms on gallstone in patients with HBV-related liver failure is still unknown. Our studies showed that there were no significant differences in the frequency of UGT1A1*6 between GRHF (32.4%) and CTRL, HBV (29.6%) and CTRL (26.5%), GFHF (34.3%) and HBV, GRHF and HBV, or GRHF and GFHF, suggesting that this variant was not associated with cholelithiasis observed in HBV-related liver failure. By contrast, Chaouch et al. showed that allele A and genotype AA are significantly related to a decreased risk of gallstone in Tunisian patients with cholelithiasis, suggesting that c.211G>A seems to be linked with a protective effect against gallstone [37]. Nonetheless, the different conclusions may be ascribed to the small sample size.

UGT1A1*27 is described as a substitution of cytosine by adenine, changing amino acid 229 from proline to glycine, and its mutations may have phenotypically severe jaundice classified as Crigler–Najjar syndrome [38]. Here, we presented that the frequency of UGT1A1*27 in GRHF group was 8.8%, which was obviously higher than that in the CTRL group (0.0%). The odds ratio of UGT1A1*27 was 25.22, showing that UGT1A1*27 increased the risk of gallstone in HBV-related hepatic failure. The frequencies of the genotype CA and allele A of UGT1A1*27 in GRHF were significantly higher than those of CTRL or HBV groups. This may imply that gallstone and hepatic failure are related to UGT1A1*27. A recent study from Malaysia revealed that Jaundiced neonates with severe neonatal hyperbilirubinemia were detected with genetic mutant UGT1A1*27 (c.686C>A) [33]. UGT1A1*27 mutation was detected in the patient with hyperbilirubinemia and his mother [27]. Clinical data of Korean patients with hyperbilirubinemia showed that UGT1A1*27 allele was significantly different between patients and healthy individuals [39]. On the contrary, UGT1A1*27 variants were not observed in Indian patients with neonatal hyperbilirubinemia, which may indicate the low frequency of c.686C>A in the study cohort [36].

TATA sequence polymorphism in *UGT1A1* is strongly associated with glucuronidation rates of bilirubin [28]. It has been found that [TA]7 and [TA]8 variants are associated with high levels of bilirubin and increased risk of cholelithiasis in Tunisia [37]. A statistically significant association has been confirmed between allele ([TA]7) or phenotypes ([TA]6/[TA]7 and [TA]7/[TA]7) and cholelithiasis in Kuwaiti subjects with hemoglobinopathy [40]. A case–control study has suggested that chronic hepatitis C patients with increased bilirubin levels have a high frequency of UGT1A1*28 [24]. However, few studies have showed that allele ([TA]7) and genotypes ([TA]6/[TA]7 and [TA]7/[TA]7) are risk factors for cholelithiasis in liver failure caused by HBV. In our study, we found that the frequency of UGT1A1*28 variants in the CTRL group (15.9%) was lower than that in the GRHF group (35.3%). The odds ratio of UGT1A1*28 variants was 2.88 with a 95% confidence interval of 1.21–6.84, indicating that UGT1A1*28 variants were associated with cholecystolithiasis in HBV-related liver failure. By contrast, genotypes [TA]6/[TA]7 and [TA]7/[TA]7 are not significantly associated with gallstone phenotype in patients with HBV-related liver failure. Given the small sample size, this result should be viewed with caution.

UGT1A1*60 is a common variant located in the promoter region, which is a risk factor for Gilbert syndrome [29]. Sugatani et al. found that compound heterozygosity and homozygosity for mutations in the promoter of *UGT1A1* gene ([TA]6>[TA]7 and c.-3279T>G) are associated with the hyperbilirubinemia in most patients with Gilbert’s syndrome [29]. For neonatal jaundice in the Malay population, c.-3279T>G, in the promoter of *UGT1A1* gene, is also recognized as a risk factor [41]. Besides, the transcriptional activity of the c.-3279G allele was decreased compared with c.-3279T allele [41]. Homozygous mutation of c.-3279T>G is associated with a high level of total serum bilirubin, which represents a significant risk factor for the development of neonatal hyperbilirubinemia [42]. Besides, homozygosity of c.-3279T>G allele combined with [TA]7 heterozygous genotype is associated with pediatric mild hyperbilirubinemia [43]. It was confirmed that A–T haplotype increases the risk of hyperbilirubinemia instead of c.-3279T>G and c.-3156G>A variants alone in an Iranian population [44]. However, the frequency of UGT1A1*60 is not significantly different between GRHF and CTRL groups, implying that this variant was not associated gallstone in patients with HBV-related liver failure.

To further investigate the combined effects of SNPs in *UGT1A1* on the development of gallstone- and HBV-related liver failure, haplotype analysis was carried out. Healthy participants showed a high frequency of the haplotype G-C-[TA]-G compared with GRHF patients (15.3%), revealing an association of the haplotype G-C-[TA]-G to healthy individuals. The haplotype G-C-[TA]7-T was significantly associated with GRHF compared to GFHF patients, indicating that this haplotype might be the causative factor for the development of GRHF. This result revealed that haplotype G-G-[TA]7-T may be associated with gallstone in HBV-related hepatic failure. Besides the genetic factors, cirrhotic individuals are sensitive to bile acid composition and bile nucleation, which leads to the generation of biliary stones and symptomatic cholelithiasis [45,46]. Although this study first reports the effects of *UGT1A1* single-nucleotide polymorphism on gallstone-related liver failure caused by HBV, these results should be seriously viewed because of the small sample size, or be confirmed by *in vivo* experiments.

## 5 Conclusions

In total, this study broadens the knowledge concerning the association between gallstone and *UGT1A1* variations in patients with HBV-related liver failure. Patients with GFHF showed increased levels of serum total bilirubin, direct bilirubin, and indirect bilirubin. UGT1A1*27 and UGT1A1*28 showed a strong association with GRHF. Haplotype G-C-[TA]7-T (UGT1A1*6, UGT1A1*27, UGT1A1*28, and UGT1A1*60) was significantly associated with GRHF patients compared with GFHF patients. Based on the finding that UGT1A1*27 and UGT1A1*28 variants were significantly observed in gallstone-related liver failure induced by HBV, our project highlights the value of *UGT1A1* genotypes in genetic testing and pathogenetic studies. However, further expansion of the study population is required because statistical bias is caused by the low available population from the determined time set.

## Supplementary Material

Supplementary Table
