# Supplementary material for: Association of gallstone and polymorphisms of UGT1A1*27 and UGT1A1*28 in patients with hepatitis B virus-related liver failure
Source: Open Med (Wars). 2022 Sep 6;17(1):1455–65. doi: 10.1515/med-2022-0549 (PMC9449690; doi:10.1515/med-2022-0549)
Supplement: Supplementary Table [file med-2022-0549-sm.pdf]

Supplementary materials

Table S1: Odds ratio and 95% CI for gallbladder stone-related hepatic failure associated with UGT1A1 (NM_000463) variants

	**Polymorphisms**			
	UGT1A1*6	***UGT1A1*27***	***UGT1A1*28***	***UGT1A1*60***
Variant	c.211G>A	c.686C>A	[TA]6>[TA]7	c.-3279T>G
Amino acid change	p.Gly71Arg	p.Pro229Glu	—	—
SNP ID	rs4148323	rs35350960	rs3064744	rs4124874
Location	Exon 1	Exon 1	Promoter	Promoter
Position	GRCh38.p13	GRCh38.p13	GRCh38.p13	GRCh38.p13
Type of variant	Missense	Missense	Upstream Transcript	Upstream Transcript
CTRL (*n*, freq)	30, 26.5%	0, 0.0%	18, 15.9%	67, 59.3%
GRHF (*n*, freq)	11, 32.4%	3, 8.8%	12, 35.3%	18, 52.9%
Fisher's *p*-value	0.5188	0.0115	0.0266	0.5556
OR[95% CI]	1.32[0.58,3.04]	25.22 [1.27,502]	2.88 [1.21,6.84]	0.77 [0.36,1.67]
CTRL (*n*, freq)	30, 26.5%	0, 0.0%	18, 15.9%	67, 59.3%
HBV (*n*, freq)	16, 29.6%	0, 0.0%	13, 24.1%	29, 53.7%
Fisher's *p*-value	0.7131	1.0000	0.2099	0.5080
OR[95% CI]	1.17 [0.57,2.39]	2.08 [0.04,106.5]	1.67 [0.75,3.73]	0.80 [0.41,1.53]
HBV (*n*, freq)	16, 29.6%	0, 0.0%	13, 24.1%	29, 53.7%
GFHF (*n*, freq)	46, 34.3%	6, 4.5%	35, 26.1%	75, 56.0%
Fisher's *p*-value	0.6087	0.1846	0.8545	0.8714
OR[95% CI]	1.24 [0.63,2.46]	5.51 [0.31,99.66]	1.12 [0.54,2.32]	1.10 [0.58,1.07]
HBV (*n*, freq)	16, 29.6%	0, 0.0%	13, 24.1%	29, 53.7%
GRHF (*n*, freq)	11, 32.4%	3, 8.8%	12, 35.3%	18, 52.9%
Fisher's *p*-value	0.8158	0.0545	0.3325	1.0000
OR[95% CI]	1.14 [0.45,2.87]	12.11 [0.61,242.3]	1.72 [0.67,4.41]	0.97 [0.41,2.29]
GFHF (*n*, freq)	46, 34.3%	6, 4.5%	35, 26.1%	75, 56.0%
GRHF (*n*, freq)	11, 32.4%	3, 8.8%	12, 35.3%	18, 52.9%
Fisher's *p*-value	1.0000	0.3884	0.2922	0.8473
OR[95% CI]	0.91 [0.41,2.04]	2.07 [0.49,8.72]	1.54 [0.69,3.44]	0.89 [0.42,1.88]

CI, confidence interval; UGT1A1, UDP-glucuronosyltransferase 1A1; SNP, single nucleotide polymorphism; OR, odds ratio; HBV, hepatitis B virus. *p*-values were calculated using Fisher's exact test, and OR was calculated by adding 0.5 to the value 0. CTRL group, healthy individuals without HBV infection, gallstone, or hepatic failure; HBV group, patients with HBV infection; GFHF, patients with gallstone-free hepatic failure and HBV infection; GRHF, patients with gallstone-related hepatic failure and HBV infection. CTRL (n = 113), HBV (n = 54), GFHF (n = 134), GRHF (n = 34).

Table S2: Genotype distribution and allele frequencies of 4 SNPs in UGT1A1 gene with the development of hepatic failure associated with gallbladder stone

**Polymorphisms**	**Genotype (*n*, frequency)**			**Allele (*n*, frequency)**	
UGT1A1*6	**GG**	**GA**	**AA**	**G**	**A**
CTRL (*n*, freq)	83, 73.5%	27, 23.9%	3, 2.7%	193, 85.4%	33, 14.6%
GRHF (*n*, freq)	23, 67.6%	9, 26.5%	2, 5.9%	55, 80.9%	13, 19.1%
Fisher's *p*-value	—	0.6527	0.3140	—	0.4460
OR[95%CI]	1	1.20 [0.50,2.91]	2.41 [0.38,15.27]	1	1.38 [0.68,2.81]
CTRL (*n*, freq)	83, 73.5%	27, 23.9%	3, 2.7%	193, 85.4%	33, 14.6%
HBV (*n*, freq)	38, 70.4%	15, 27.8%	1, 1.9%	91, 84.3%	17, 15.7%
Fisher's *p*-value	—	0.7026	1.0000	—	0.8699
OR[95%CI]	1	1.21 [0.58,2.54]	0.73 [0.07,7.23]	1	1.09 [0.58,2.06]
HBV (*n*, freq)	38, 70.4%	15, 27.8%	1, 1.9%	91, 84.3%	17, 15.7%
GFHF (*n*, freq)	88, 65.7%	41, 30.6%	5, 3.7%	217, 81.0%	51, 19.0%
Fisher's *p*-value	—	0.7251	0.6696	—	0.5539
OR[95%CI]	1	1.18 [0.58,2.39]	2.16 [0.24,19.12]	1	1.26 [0.69,2.30]
HBV (*n*, freq)	38, 70.4%	15, 27.8%	1, 1.9%	91, 84.3%	17, 15.7%
GRHF (*n*, freq)	23, 67.6%	9, 26.5%	2, 5.9%	55, 80.9%	13, 19.1%
Fisher's *p*-value	—	1.0000	0.5554	—	0.6810
OR[95%CI]	1	0.99 [0.37,2.63]	3.30 [0.28,38.53]	1	1.27 [0.57,2.81]
GFHF (*n*, freq)	88 (65.7%)	41 (30.6)	5 (3.7%)	217 (81.0%)	51 (19.0%)
GRHF (*n*, freq)	23, 67.6%	9, 26.5%	2, 5.9%	55, 80.9%	13, 19.1%
Fisher's *p*-value	—	0.8317	0.6386	—	1.0000
OR[95% CI]	1	0.84 [0.36,1.98]	1.53 [0.28,8.41]	1	1.01 [0.51,1.98]
*UGT1A1*27*	CC	CA	AA	C	A
CTRL (*n*, freq)	113, 100%	0, 0.0%	0, 0.0%	226, 100.0%	0, 0.0%
GRHF (*n*, freq)	31, 91.2%	3, 8.8%	0, 0.0%	65, 95.6%	3, 4.4%
Fisher's *p*-value	—	0.0115	1.000	—	0.0120
OR[95%CI]	1	25.22 [1.27,502]	3.60 [0.07,185]	1	24.21 [1.23,475]
CTRL (*n*, freq)	113, 100%	0, 0.0%	0, 0.0%	226, 100.0%	0, 0.0%
HBV (*n*, freq)	54, 100%	0, 0.0%	0, 0.0%	108, 100.0%	0, 0.0%
Fisher's *p*-value	—	1.0000	1.0000	—	1.0000
OR[95%CI]	1	2.08 [0.04,107]	2.08 [0.04,107]	1	2.09 [0.04,106]
HBV (*n*, freq)	54, 100%	0, 0.0%	0, 0.0%	108, 100.0%	0, 0.0%
GFHF (*n*, freq)	128, 95.5%	6, 4.5%	0, 0.0%	262, 97.8%	6, 2.2%
Fisher's *p*-value	—	0.1846	1.0000	—	0.1883
OR[95%CI]	1	5.51 [0.31,99.66]	0.42 [0.01,21.67]	1	5.37 [0.30,96.28]
HBV (*n*, freq)	54, 100.0%	0, 0.0%	0, 0.0%	108, 100.0%	0, 0.0%
GRHF (*n*, freq)	31, 91.2%	3, 8.8%	0, 0.0%	65, 95.6%	3, 4.4%
Fisher's *p*-value	—	0.0264	1.0000	—	0.0277
OR[95%CI]	1	12.11 [0.61,242]	1.73 [0.03,89.44]	1	11.60 [0.59,228]
GFHF (*n*, freq)	128, 95.5%	6, 4.5%	0, 0.0%	262, 97.8%	6, 2.2%

(*Continued*)

Table S2: *Continued*

**Polymorphisms**	**Genotype (*n*, frequency)**			**Allele (*n*, frequency)**	
UGT1A1*6	**GG**	**GA**	**AA**	**G**	**A**
GRHF (*n*, freq)	31, 91.2%	3, 8.8%	0, 0.0%	65, 95.6%	3, 4.4%
Fisher's *p*-value	—	0.3884	1.0000	—	0.3937
OR[95% CI]	1	2.07 [0.49,8.72]	4.08 [0.08,209.8]	1	2.02 [0.49,8.28]
*UGT1A1*28*	[TA]6[TA]6	[TA]6[TA]7	[TA]7[TA]7	[TA]6	[TA]7
CTRL (*n*, freq)	95, 84.1%	18, 15.9%	0, 0.0%	208, 92.0%	18, 8.0%
GRHF (*n*, freq)	22, 64.7%	11, 32.4%	1, 2.9%	55, 80.9%	13, 19.1%
Fisher's *p*-value	—	0.0448	0.1949	—	0.0129
OR[95%CI]	1	2.64 [1.09,6.38]	12.73 [0.50,323]	1	2.73 [1.26,5.92]
CTRL (*n*, freq)	95, 84.1%	18, 15.9%	0, 0.0%	208, 92.0%	18, 8.0%
HBV (*n*, freq)	41, 75.9%	12, 22.2%	1, 1.9%	94, 87.0%	14, 13.0%
Fisher's *p*-value	—	0.3868	0.3066	—	0.1656
OR[95%CI]	1	1.55[0.68,3.50]	6.90[0.28,173]	1	1.72[0.82,3.61]
HBV (*n*, freq)	41, 75.9%	12, 22.2%	1, 1.9%	94, 87.0%	14, 13.0%
GFHF (*n*, freq)	99, 73.9%	35, 26.1%	0, 0.0%	233, 86.9%	35, 13.1%
Fisher's *p*-value	—	0.7101	0.2979	—	1.0000
OR[95%CI]	1	1.21 [0.57,2.56]	0.14 [0.01,3.49]	1	1.01 [0.52,1.96]
HBV (*n*, freq)	41, 75.9%	12, 22.2%	1, 1.9%	94, 87.0%	14, 13.0%
GRHF (*n*, freq)	22, 64.7%	11, 32.4%	1, 2.9%	55, 80.9%	13, 19.1%
Fisher's *p*-value	—	0.3213	1.0000	—	0.2890
OR[95%CI]	1	1.71 [0.65,4.50]	1.86 [0.11,31.28]	1	1.59 [0.70,3.62]
GFHF (*n*, freq)	99 (73.9%)	35 (26.1%)	0 (0.0%)	233 (86.9%)	35 (13.1%)
GRHF (*n*, freq)	22, 64.7%	11, 32.4%	1, 2.9%	55, 80.9%	13, 19.1%
Fisher's *p*-value	—	0.3945	0.1885	—	0.2431
OR[95% CI]	1	1.41 [0.62,3.21]	13.27 [0.52,337]	1	1.57 [0.78,3.17]
*UGT1A1*60*	TT	TG	GG	T	G
CTRL (*n*, freq)	46, 40.7%	53, 46.9%	14, 12.4%	145, 64.2%	81, 35.8%
GRHF (*n*, freq)	16, 47.1%	14, 41.2%	4, 11.8%	46, 67.6%	22, 32.4%
Fisher's *p*-value	—	0.5379	1.0000	—	0.6647
OR[95%CI]	1	0.76 [0.33,1.72]	0.82 [0.24,2.86]	1	0.86 [0.48,1.52]
CTRL (*n*, freq)	46, 40.7%	53, 46.9%	14, 12.4%	145, 64.2%	81, 35.8%
HBV (*n*, freq)	25, 46.3%	23, 42.6%	6, 11.1%	73, 67.6%	35, 32.4%
Fisher's *p*-value	—	0.5985	0.7921	—	0.6233
OR[95%CI]	1	0.80 [0.40,1.59]	0.79 [0.27,2.31]	1	0.86 [0.53,1.40]
HBV (*n*, freq)	25, 46.3%	23, 42.6%	6, 11.1%	73, 67.6%	35, 32.4%
GFHF (*n*, freq)	59, 44.0%	63, 47.0%	12, 9.0%	181, 67.5%	87, 32.5%
Fisher's *p*-value	—	0.7342	0.7821	—	1.0000
OR[95%CI]	1	1.16 [0.59,2.27]	0.85 [0.29,2.51]	1	1.00 [0.62,1.62]
HBV (*n*, freq)	25, 46.3%	23, 42.6%	6, 11.1%	73, 67.6%	35, 32.4%

(*Continued*)

Table S2: *Continued*

**Polymorphisms**	**Genotype (*n*, frequency)**			**Allele (*n*, frequency)**	
UGT1A1*6	**GG**	**GA**	**AA**	**G**	**A**
GRHF (*n*, freq)	16, 47.1%	14, 41.2%	4, 11.8%	46, 67.6%	22, 32.4%
Fisher's *p*-value	—	1.0000	1.0000	—	1.0000
OR[95%CI]	1	0.95 [0.38,2.37]	1.04 [0.25,4.28]	1	0.998 [0.52,1.91]
GFHF (*n*, freq)	59, 44.0%	63, 47.0%	12, 9.0%	181 (67.5%)	87 (32.5%)
GRHF (*n*, freq)	16, 47.1%	14, 41.2%	4, 11.8%	46, 67.6%	22, 32.4%
Fisher's *p*-value	—	0.6862	0.7457	—	1.0000
OR[95% CI]	1	0.82 [0.37,1.83]	1.23 [0.35,4.33]	1	0.995 [0.56,1.76]

p-values were calculated using Fisher's exact test, and OR was calculated by adding 0.5 to the value 0. UGT1A1, UDP-glucuronosyltransferase 1A1; SNP, single nucleotide polymorphism; HBV, hepatitis B virus. CTRL group, healthy individuals without HBV infection, gallstone, or hepatic failure; HBV group, patients with HBV infection; GFHF, patients with gallstone-free hepatic failure and HBV infection; GRHF, patients with gallstone-related hepatic failure and HBV infection. CTRL (n = 113), HBV (n = 54), GFHF (n = 134), GRHF (n = 34).
